# MG132, a proteasome inhibitor, induces human pulmonary fibroblast cell death via increasing ROS levels and GSH depletion

**DOI:** 10.3892/or.2012.1642

**Published:** 2012-01-19

**Authors:** WOO HYUN PARK, SUHN HEE KIM

**Affiliations:** Department of Physiology, Medical School, Research Institute for Endocrine Sciences, Chonbuk National University, Jeonju 561-180, Republic of Korea

**Keywords:** MG132, proteasome, cell death, human pulmonary fibroblast, reactive oxygen species

## Abstract

MG132 as a proteasome inhibitor can induce apoptotic cell death in lung cancer cells. However, little is known about the toxicological cellular effects of MG132 on normal primary lung cells. Here, we investigated the effects of N-acetyl cysteine (NAC) and vitamin C (well known antioxidants) or L-buthionine sulfoximine (BSO; an inhibitor of GSH synthesis) on MG132-treated human pulmonary fibroblast (HPF) cells in relation to cell death, reactive oxygen species (ROS) and glutathione (GSH). MG132 induced growth inhibition and death in HPF cells, accompanied by the loss of mitochondrial membrane potential (MMP; ΔΨ_m_). MG132 increased ROS levels and GSH-depleted cell numbers in HPF cells. Both antioxidants, NAC and vitamin C, prevented growth inhibition, death and MMP (ΔΨ_m_) loss in MG132-treated HPF cells and also attenuated ROS levels in these cells. BSO showed a strong increase in ROS levels in MG132-treated HPF cells and slightly enhanced the growth inhibition, cell death, MMP (ΔΨ_m_) loss and GSH depletion. In addition, NAC decreased anonymous ubiquitinated protein levels in MG132-treated HPF cells. Furthermore, superoxide dismutase (SOD) 2, catalase (CTX) and GSH peroxidase (GPX) siRNAs enhanced HPF cell death by MG132, which was not correlated with ROS and GSH level changes. In conclusion, MG132 induced the growth inhibition and death of HPF cells, which were accompanied by increasing ROS levels and GSH depletion. Both NAC and vitamin C attenuated HPF cell death by MG132, whereas BSO slightly enhanced the death.

## Introduction

Reactive oxygen species (ROS) include hydrogen peroxide (H_2_O_2_), the superoxide anion (O_2_^•−^) and the hydroxyl radical (^•^OH). ROS are involved in the regulation of many important cellular events, including transcription factor activation, gene expression, differentiation and cell proliferation (1,2). ROS are generated as by-products of mitochondrial respiration or by oxidases, such as the nicotine adenine diphosphate (NADPH) oxidase and the xanthine oxidase (XO) (3). A change in the redox state of tissues or cells alters the generation or metabolism of ROS. The principal metabolic pathways involved in redox defense, include superoxide dismutases (SOD), which involve 3 isoforms, the extracellular (SOD3), cytoplasmic (SOD1), and the mitochondrial (SOD2) isoforms (4), which metabolize O_2_^•−^ to H_2_O_2_. Further metabolism by peroxidases, including catalase (CAT) and glutathione (GSH) peroxidase (GPX), yields O_2_ and H_2_O (5). Cells have various antioxidant systems to manage their redox state, which is important for their survival. The thioredoxin (TXN) system consists of TXN, TXN reductase and NADPH and is critically involved in maintaining cellular redox homeostasis (6). TXN as a thiol reductase is a potent antioxidant and acts as a scavenger of ROS (6). Excessive production of ROS can be induced by endogenous and/or exogenous sources, which then initiates events that lead to cell death depending on the cell type (7–9).

The ubiquitin-dependent proteasomal system presents the foremost non-lysosomal corridor through which intracellular proteins involved in cell cycling, proliferation, differentiation and apoptosis are degraded in eukaryotic cells (10,11). Transformed cells including cancer cells accumulate more misfolded/mutated/damaged proteins due to the elevated replication rate of malignant cells (12). Thus, these cells can be much more susceptible to proteasome inhibition than normal cells. Apoptosis in cancer cells is closely connected with the activity of the ubiquitin/proteasome pathways (13,14). Accordingly, the inhibition of proteasome function has emerged as a useful strategy to control apoptosis. The peptide aldehyde MG132 (carbobenzoxy-Leu-Leu-leucinal) efficiently prevents the proteolytic activity of the proteasome complex (15). Various proteasome inhibitors including MG132 have been demonstrated to stimulate apoptotic cell death through the induction of ROS (16,17). ROS formation and GSH depletion by proteasome inhibitors may trigger mitochondrial dysfunction and subsequent cytochrome C release, which can lead to cell death (18,19). The mechanism underlying ROS generation after inhibition of the proteasome is still imprecise.

Lung cancer is a main cause of cancer death in developed countries. Various novel remedial strategies including new drug development are currently under consideration due to intrinsic or acquired resistance and toxicity of conventional drugs (20). Specifically, drugs that aim at specific intracellular pathways related to the distinctive properties of cancer cells continue to be developed. Recently, it has been reported that a proteasome inhibitor bortezomib (PS-341, Velcade) inhibits lung cancer cells (21,22). The toxicological mechanism of MG132 in lung cancer cells has not been fully understood. We recently demonstrated that MG132 reduced the growth of Calu-6 and A549 lung cancer cells via apoptosis and GSH depletion (23,24). On the other hand, little is known about the cellular effects of MG132 on normal primary lung cells in relation to cell death. Because we observed that MG132 induced the growth inhibition and death in human pulmonary fibroblast (HPF) cells via a caspase-independent manner (unpublished data), in the present study, we investigated the effects of N-acetyl cysteine (NAC) and vitamin C (well known antioxidants) or L-buthionine sulfoximine (BSO; an inhibitor of GSH synthesis) (25) on MG132-treated HPF cells in relation to cell growth, death, ROS and GSH levels. Furthermore, we examined the effects of antioxidant-related siRNAs on cell death, ROS and GSH levels in MG132-treated HPF cells.

## Materials and methods

### Cell culture

The human pulmonary fibroblast (HPF) cells from PromoCell GmbH (Heidelberg, Germany) were maintained in humidified incubator containing 5% CO_2_ at 37°C. HPF cells were cultured in RPMI-1640 supplemented with 10% fetal bovine serum (FBS) and 1% penicillin-streptomycin (Gibco-BRL, Grand Island, NY). HPF cells were used between passages four and eight.

### Reagents

MG132 was purchased from Calbiochem (San Diego, CA) and was dissolved in dimethyl sulfoxide (DMSO; Sigma-Aldrich, St. Louis, MO) solution buffer. NAC and BSO were obtained from Sigma-Aldrich. NAC was dissolved in 20 mM HEPES (pH 7.0) buffer. BSO was dissolved in water. Vitamin C purchased from Riedel-de Haen (Hannover, Germany) was also dissolved in water. Based on previous studies (26,27) cells were pretreated with 2 mM NAC or 10 μM BSO or 0.4 mM vitamin C for 1 h prior to MG132 treatment. DMSO (0.2%) was used as a control vehicle and it did not appear to affect cell growth or death.

### Detection of intracellular ROS and O_2_^•−^ levels

Intracellular ROS levels were detected by means of an oxidation-sensitive fluorescent probe dye, 2′,7′-dichlorodihydrofluorescein diacetate (H_2_DCFDA, Ex/Em of 495 nm/529 nm; Invitrogen Molecular Probes, Eugene, OR) as previously described (28). H_2_DCFDA is poorly selective for superoxide anion radical (O_2_^•−^). On the other hand, dihydroethidium (DHE) (Ex/Em of 518 nm/605 nm; Invitrogen Molecular Probes) is a fluorogenic probe that is highly selective for O_2_^•−^ (28). Mitochondrial O_2_^•−^ levels were detected using the MitoSOX™ Red mitochondrial O_2_^•−^ indicator (Ex/Em of 510 nm/580 nm; Invitrogen Molecular Probes) as previously described (28). In brief, 1×10^6^ cells in 60-mm culture dish (Nunc) were incubated with the indicated doses of MG132 with or without NAC, BSO, vitamin C or antioxidant-related siRNA duplexes for 24 h. Cells were then washed in PBS and incubated with 20 μM H_2_DCFDA, 20 μM DHE or 5 μM MitoSOX Red at 37°C for 30 min. DCF, DHE and MitoSOX Red fluorescence intensities were detected using a FACStar flow cytometer (Becton-Dickinson, Franklin Lakes, NJ). ROS and O_2_^•−^ levels were expressed as mean fluorescence intensity (MFI), using the CellQuest software (Becton-Dickinson).

### Detection of intracellular glutathione (GSH) levels

Cellular GSH levels were analyzed using a 5-chloromethylfluorescein diacetate dye (CMFDA, Ex/Em of 522 nm/595 nm; Invitrogen Molecular Probes) as previously described (28). In brief, 1×10^6^ cells in 60-mm culture dishes (Nunc) were incubated with the indicated doses of MG132 with or without NAC, BSO, vitamin C or antioxidant-related siRNA duplexes for 24 h. Cells were then washed with PBS and incubated with 5 μM CMFDA at 37°C for 30 min. CMF fluorescence intensity was determined using a FACStar flow cytometer (Becton-Dickinson). Negative CMF staining (GSH depleted) cells were expressed as the percent of (−) CMF cells.

### Cell growth inhibition assays

The effect of drugs on HPF cell growth was determined by the 3-(4,5-dimethylthiazol-2-yl)-2,5-diphenyltetrazolium bromide (MTT) assay as previously described (29). In brief, 5×10^3^ cells/well were seeded in 96-well microtiter plates (Nunc). After exposure to the indicated doses of MG132 with or without NAC, BSO or vitamin C for 24 h, 20 μl of MTT solution [2 mg/ml in phosphate-buffered saline (PBS)] were added to each well of the 96-well plates. The plates were incubated for 4 additional hours at 37°C. Media in plates were withdrawn by pipetting and 200 μl of DMSO was added to each well to solubilize the formazan crystals. Optical density was measured at 570 nm using a microplate reader (SpectraMAX 340, Molecular Devices Co., Sunnyvale, CA).

### Annexin-V/PI staining for cell death detection

Apoptosis was determined by staining cells with Annexin-V-fluorescein isothiocyanate (FITC, Ex/Em of 488 nm/519 nm; Invitrogen Molecular Probes) and propidium iodide (PI, Ex/Em of 488 nm/617 nm; Sigma-Aldrich). In brief, 1×10^6^ cells in 60-mm culture dishes (Nunc) were incubated with the indicated doses of MG132 with or without NAC, BSO, vitamin C or antioxidant-related siRNA duplex for 24 h. Cells were washed twice with cold PBS and then resuspended in 500 μl binding buffer (10 mM HEPES/NaOH pH 7.4, 140 mM NaCl, 2.5 mM CaCl_2_) at a concentration of 1×10^6^ cells/ml. Annexin-V-FITC (5 μl) and PI (1 μg/ml) were then added to these cells, which were analyzed with a FACStar flow cytometer (Becton-Dickinson). Viable cells were negative for both PI and Annexin-V; apoptotic cells were positive for Annexin-V and negative for PI, whereas late apoptotic dead cells displayed both high Annexin-V and PI labeling. Non-viable cells, which underwent necrosis, were positive for PI and negative for Annexin-V.

### Measurement of MMP (ΔΨ_m_)

MMP (ΔΨ_m_) levels were measured using a rhodamine 123 fluorescent dye (Sigma-Aldrich; Ex/Em of 485 nm/535 nm) as previously described (30). In brief, 1×10^6^ cells in 60-mm culture dishes (Nunc) were incubated with the indicated doses of MG132 with or without NAC, BSO or vitamin C for 24 h. Cells were washed twice with PBS and incubated with rhodamine 123 (0.1 μg/ml) at 37°C for 30 min. Rhodamine 123 staining intensity was determined by flow cytometry (Becton-Dickinson). An absence of rhodamine 123 from cells indicated the loss of MMP (ΔΨ_m_) in HPF cells.

### Western blot analysis

The patterns of ubiquitinated proteins were evaluated using Western blot analysis. In brief, 1×10^6^ cells in 60-mm culture dish (Nunc) were incubated with 10 μM MG132 with or without NAC for 24 h. The cells were then washed in PBS and suspended in five volumes of lysis buffer (20 mM HEPES, pH 7.9, 20% glycerol, 200 mM KCl, 0.5 mM EDTA, 0.5% NP-40, 0.5 mM DTT and 1% protease inhibitor cocktail). Supernatant protein concentrations were determined using the Bradford method. Samples containing 40 μg total protein were resolved by 12.5% SDS-PAGE gels, transferred to Immobilon-P PVDF membranes (Millipore, Billerica, MA) by electroblotting and then probed with anti-ubiquitin and anti-β-actin antibodies (Santa Cruz Biotechnology, Santa Cruz, CA). Membranes were incubated with horseradish peroxidase-conjugated secondary antibodies. Blots were developed using an ECL kit (Amersham, Arlington Heights, IL).

### Transfection of cells with antioxidant-related siRNAs

Gene silencing of SOD1, SOD2, CAT, GPX, TXN was performed as previously described (31). The siRNA duplexes consisted of a non-specific control siRNA duplex [5′-CCUACGCCACCAAUUUCGU(dTdT)-3′], the SOD1 [5′-GAAAACACGGUGGGCCAAA(dTdT)-3′], the SOD2 [5′-CUGGGAGAAUGUAAC UGAA (dTdT)-3′], the CAT [5′-CACUGAUUUCACAACAGAU (dTdT)-3′], the GPX [5′-CAAGCUCAUCACCUGGUCU (dTdT)-3′] and the TXN [5′-GCAUGCCAACAUUCCAGUU (dTdT)-3′] siRNA duplexes which were purchased from the Bioneer Corp. (Daejeon, South Korea). In brief, 2.5×10^5^ cells in 6-well plates (Nunc) were incubated in RPMI-1640 supplemented with 10% FBS. The next day, cells (30–40% confluence) in each well were transfected with the control or each siRNA duplex [80 picomoles in Opti-MEM (Gibco-BRL)] using Lipofectamine 2000, according to the manufacturer's instructions (Invitrogen, Brandford, CT). Two days later, cells were treated with or without 30 μM MG132 for 24 additional hours. The transfected cells were collected and used for the measurement of Annexin-V-FITC/PI staining cells, ROS and GSH depletion levels.

### Statistical analysis

The results represent the mean of at least 3 independent experiments (mean ± SD). The data were analyzed using Instat software (GraphPad Software, Inc., San Diego, CA). The Student's t-test or one-way analysis of variance (ANOVA) with post-hoc analysis using Tukey's multiple comparison test was used for parametric data. Statistical significance was defined as P<0.05.

## Results

### MG132 alters ROS and GSH levels in HPF cells

**Figure 1:**
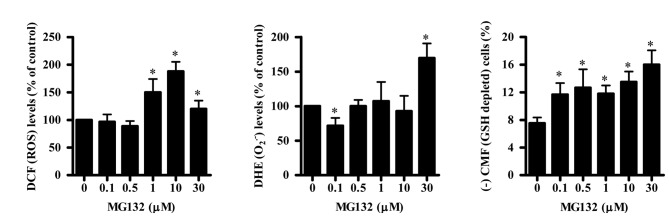
Effects of MG132 on ROS and GSH levels in HPF cells. Exponentially growing cells were treated with the indicated amounts of MG132 for 24 h. ROS and GSH levels were measured with a FACStar flow cytometer. (A and B) Graphs indicate ROS (as determined by DCF) levels (%) (A) and DHE (O_2_^•−^) levels (%) compared with control cells (B). (C) Graph shows the percent of (−) CMF (GSH-depleted) cells. ^*^P<0.05 compared with the control group.

To assess intracellular ROS and GSH levels in MG132-treated HPF cells, we used (0.1–30 μM) of MG132 based on our unpublished findings that 0.5–30 μM MG132 dose-dependently inhibited the growth of HPF cells with an IC_50_ of ~20 μM at 24 h. As shown in Fig. 1A, intracellular ROS (DCF) levels were not altered in HPF cells treated with 0.1 or 0.5 μM MG132 but were increased in 1–30 μM MG132-treated HPF cells. Intracellular O_2_^•−^ (DHE) level was decreased in HPF cells treated with 0.1 μM MG132 and was not significantly changed by 0.5, 1 and 10 μM MG132 (Fig. 1B). An increase in O_2_^•−^ levels was observed in 30 μM MG132-treated HPF cells (Fig. 1B). In relation to GSH levels in MG132-treated HPF cells, MG132 increased the number of GSH-depleted HPF cells at 24 h in a dose-dependent manner as compared with those of the control cells (Fig. 1C).

### NAC, vitamin C or BSO influences the growth inhibition and death of MG132-treated HPF cells

**Figure 2:**
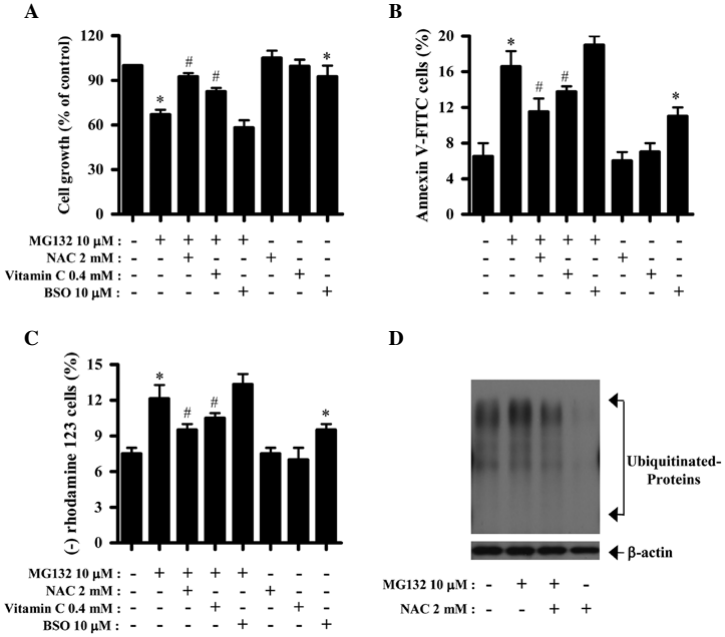
Effects of NAC, vitamin C or BSO on cell growth, cell death and MMP (ΔΨ_m_) in MG132-treated HPF cells. Exponentially growing cells were treated with 10 μM MG132 for 24 h following a 1 h of pre-incubation with 2 mM NAC, 0.4 mM vitamin C or 10 μM BSO. (A) The graph shows cell growth changes in HPF cells as assessed by the MTT assay. (B and C) Annexin-V-FITC cells and MMP (ΔΨ_m_) loss cells were measured with a FACStar flow cytometer. Graphs show the percent of Annexin V-positive staining cells (B) and rhodamine 123-negative [MMP (ΔΨ_m_) loss] cells (C). (D) Samples of protein extracts (40 μg) were resolved by SDS-PAGE gel, transferred onto PVDF membranes and immunoblotted with the indicated antibodies against ubiquitin and β-actin. ^*^P<0.05 compared with the control group. ^#^P<0.05 compared with cells treated with MG132 only.

We examined the effect of NAC, vitamin C and BSO on the growth and death of MG132-treated HPF cells. For this experiment, 10 μM MG132 was chosen as a suitable dose to examine cell growth inhibition and death in the presence or absence of NAC, vitamin C or BSO. Based on the MTT assay, 10 μM MG132 inhibited the growth of HPF cells by about 35% at 24 h (Fig. 2A). Treatment with NAC and vitamin C significantly prevented the growth inhibition by MG132 whereas BSO slightly enhanced the growth inhibition (Fig. 2A). BSO alone inhibited HPF cell growth (Fig. 2A). In relation to cell death, MG132 induced cell death in HPF cells at 24 h, as evidenced by Annexin-V staining (Fig. 2B). Both NAC and vitamin C significantly rescued HPF cells from the MG132 insult (Fig. 2B). BSO slightly increased the cell death by MG132 and this agent alone also induced cell death in HPF control cells (Fig. 2B).

Apoptosis is closely related to the collapse of MMP (ΔΨ_m_) (32). Therefore, we determined the loss of MMP (ΔΨ_m_) in MG132-treated HPF cells. Similarly to the results of Annexin-V staining; both NAC and vitamin C attenuated the loss of MMP (ΔΨ_m_) in MG132-treated HPF cells, whereas BSO mildly enhanced the loss in these cells (Fig. 2C). BSO alone induced MMP (ΔΨ_m_) loss in HPF control cells (Fig. 2C). Moreover, we observed that MG132 increased the level of anonymous ubiquitinated proteins in HPF cells (Fig. 2C). NAC showing a strong antiapoptotic effect attenuated the ubiquitinated protein levels in MG132-treated HPF cells (Fig. 2D). NAC also strongly decreased the basal ubiquitinated protein levels in HPF control cells (Fig. 2D).

### NAC, vitamin C or BSO affect ROS and GSH levels in MG132-treated HPF cells

**Figure 3:**
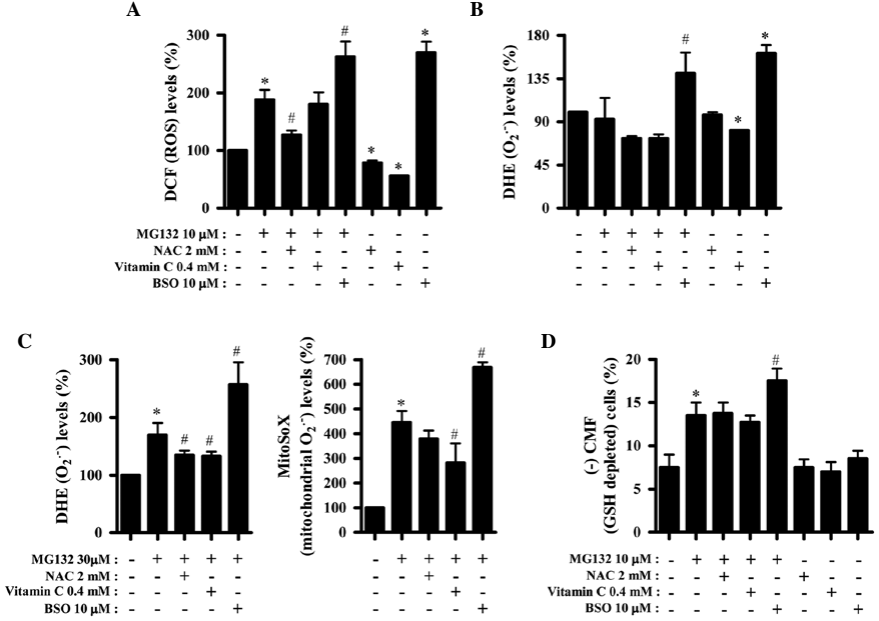
Effects of NAC, vitamin C or BSO on ROS and GSH levels in MG132-treated HPF cells. Exponentially growing cells were treated with 10 or 30 μM MG132 for 24 h following 1 h of pre-incubation with 2 mM NAC, 0.4 mM vitamin C or 10 μM BSO. ROS and GSH levels were measured with a FACStar flow cytometer. (A-C) Graphs indicate ROS (as determined by DCF) levels (%) (A), DHE (O_2_^•^) levels (%) (B and C), mitoSOX (mitochondrial O_2_^•^) levels (%) (C) compared with control cells. (D) Graph shows the percent of (−) CMF (GSH-depleted) cells. ^*^P<0.05 compared with the control group. ^#^P<0.05 compared with cells treated with MG132 only.

Next, ROS and GSH levels in HPF cells treated with 10 or 30 μM MG132with or without NAC, vitamin C or BSO were assessed. As shown in Fig. 3A, ROS (DCF) level in MG132-treated HPF cells was significantly decreased by NAC, but that was not significantly altered by vitamin C. Both NAC and vitamin C decreased basal ROS (DCF) levels in HPF control cells (Fig. 3A). In contrast, BSO strongly increased ROS (DCF) levels in MG132-treated or -untreated HPF cells (Fig. 3A). Both NAC and vitamin C seemed to decrease O_2_^•−^ levels in MG132-treated and -untreated HPF cells (Fig. 3B). However, BSO significantly increased O_2_^•−^ levels in MG132-treated or -untreated HPF cells (Fig. 3B).

In addition, we assessed the effect of NAC, vitamin C or BSO on O_2_^•−^ levels in 30 μM MG132-treated HPF cells. As shown in Fig. 3C, NAC and vitamin C attenuated O_2_^•−^ levels in these cells, but BSO strongly intensified the level. Furthermore, MitoSOX Red fluorescence levels, which specifically indicate O_2_^•−^ levels in the mitochondria, were strongly increased in 30 μM MG132-treated HPF cells after 24 h (Fig. 3C). Both NAC and vitamin C decreased the mitochondrial O_2_^•−^ levels in MG132-treated HPF cells, whereas BSO enhanced them (Fig. 3C). In relation to GSH levels, NAC did not affect the number of GSH-depleted cells among MG132-treated HPF cells, but vitamin C slightly decreased this number (Fig. 3D). BSO seemed to increase the numbers of GSH-depleted cells among MG132-treated cells (Fig. 3D). NAC, vitamin C or BSO alone did not significantly affect the percent of GSH depletion in HPF control cells (Fig. 3D).

### Antioxidant-related siRNAs affect cell death, ROS and GSH levels in MG132-treated HPF cells

**Figure 4:**
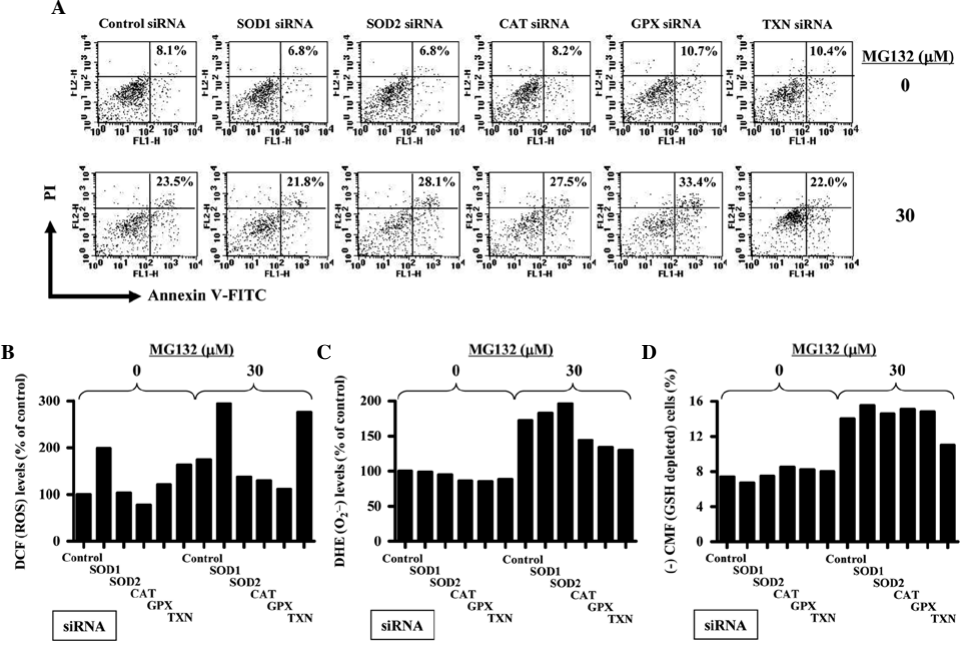
Effects of antioxidant-related siRNAs on cell death, ROS levels and GSH depletion in MG132-treated HPF cells. HPF cells (3–40% confluence) were transfected with either non-target control siRNA or each antioxidant-related siRNA. Two days later, cells were treated with 30 μM MG132 for additional 24 h. (A) Annexin V-FITC and PI cells were measured with a FACStar flow cytometer. The number (%) in each figure indicates Annexin-V-FITC positive cells regardless of PI negative and positive cells. (B and C) Graphs indicate DCF (ROS) levels (%) (B) and DHE (O_2_^•^) levels (%) (C) compared with MG132-untreated control siRNA cells. (D) Graph shows the percent of (−) CMF (GSH-depleted) cells.

Furthermore, it was determined whether antioxidant (SOD1, SOD2, CAT, GPX or TXN)-related siRNAs changed cell death, ROS and GSH levels in MG132-treated HPF cells. As shown in Fig. 4, 30 μM MG132 increased the proportion of Annexin-V-stained cells about 15% compared with that in control siRNA-treated HPF cells. Treatment with 10 μM MG132 did not clearly increase Annexin V-stained cell number in this system (data not shown). Probably, addition of Lipofectamine 2000 in the medium seemed to attenuate the biological activity of MG132. All the siRNAs of antioxidant-related proteins did not significantly alter Annexin V-stained cell number in HPF control cells for 72 h (Fig. 4A). Administration of SOD1 or TXN siRNA did not affect cell death in MG132-treated HPF cells whereas SOD2, CAT or GPX siRNA increased the Annexin-V-stained cell number in these cells (Fig. 4A). Especially, GPX siRNA treatment showed a strong pro-apoptotic effect on MG132-treated HPF cells (Fig. 4A).

In relation to ROS levels, SOD1, GPX or TXN siRNA increased ROS (DCF) levels in HPF control cells but CAT siRNA decreased the levels at 72 h (Fig. 4B). SOD1 or TXN siRNAs intensified ROS (DCF) levels in MG132-treated HPF cells whereas SOD2, CAT or GPX siRNA relatively decreased the level in these cells (Fig. 4B). CAT, GPX or TXN siRNA seemed to decrease O_2_^•−^ levels in HPF control cells (Fig. 4C). SOD2 siRNA slightly increased O_2_^•−^ levels in MG132-treated HPF cells whereas CAT, GPX or TX siRNA attenuated the level in these cells (Fig. 4C). In view of the GSH levels, all siRNAs of antioxidant-related proteins did not affect the number of GSH-depleted cells in HPF control cells for 72 h (Fig. 4D). While SOD1, SOD2, CAT or GPX siRNA did not clearly alter the GSH-depleted cell number in MG132-treated HPF cells, TNX siRNA prevented GSH deletion in these cells (Fig. 4D).

## Discussion

Various proteasome inhibitors including MG132 have been demonstrated to stimulate apoptotic cell death through the induction of ROS (16,17). Because MG132 induced growth inhibition and death in HPF cells, in the present study we focused on evaluating the molecular mechanism of MG132-induced HPF cell death in relation to ROS and GSH. According to our result, ROS level (as determined by DCF) were increased in HPF cells treated with 1, 10 or 30 μM MG132. However, O_2_^•−^ levels in HPF cells was only increased by 30 μM MG132. Thus, although MG132 generally seemed to increase intracellular ROS levels in HPF cells, it affected different ROS levels depending on its concentration. It is reported that ROS formation due to proteasome inhibitors may cause mitochondrial dysfunction and subsequent cytochrome C release, which leads to cell viability loss (18,19). The collapse of MMP (ΔΨ_m_) occurs during apoptosis (32). Correspondingly, MG132 induced the loss of MMP (ΔΨ_m_) in HPF cells. Furthermore, mitochondrial O_2_^•−^ levels in HPF cells were increased by 30 μM MG132. Although the mechanism underlying ROS generation after MG132 treatment is not clearly explained, our results suggest that increased O_2_^•−^ in MG132-treated HPF cells mainly originates from the mitochondria.

Treatment with NAC and vitamin C significantly prevented the growth inhibition of MG132-treated HPF cells and also decreased the number of Annexin-V-FITC positive cells in these cells. Both antioxidants attenuated the loss of MMP (ΔΨ_m_) in MG132-treated HPF cells. Conversely, BSO slightly enhanced growth inhibition, cell death and MMP (ΔΨ_m_) loss in MG132-treated HPF cells. These results implied that changes in ROS or GSH levels by NAC, vitamin C or BSO affected the growth inhibition and death in MG132-treated HPF cells. Thus, we assessed ROS or GSH levels in MG132-treated HPF cells in the presence or absence of NAC, vitamin C or BSO.

As expected, both antioxidants of NAC and vitamin C attenuated ROS levels including mitochondrial O_2_^•−^ levels in MG132-treated or -untreated HPF cells. BSO showing a slight enhancement in cell death and MMP (ΔΨ_m_) loss in MG132-treated HPF cells intensified ROS levels, including mitochondrial O_2_^•−^ in these cells. In addition, diethyldithiocarbamate, known to be an inhibitor of SOD (33), augmented growth inhibition, cell death, MMP (ΔΨ_m_) loss and O_2_^•−^ levels in MG132-treated HPF cells (data not shown). Therefore, MG132 seemed to induce HPF cell death through the induction of ROS. Because NAC and vitamin C individually affected different ROS levels in MG132-treated or -untreated HPF cells, each antioxidant may be exerting its effects on the prevention of MG132-induced HPF cell death via different pathways. BSO alone induced cell growth inhibition, cell death and MMP (ΔΨ_m_) loss in HPF control cells and strongly increased ROS levels. Therefore, an increased ROS by BSO treatment seemed to be tightly related to the HPF cell growth inhibition and death. Furthermore, we observed that MG132 blocked the activity of the proteasome in HPF cells, which was efficiently attenuated by NAC. These results suggest that proteasome inhibition by MG132 influences growth inhibition and death in HPF cells.

In relation to the administration of antioxidant-related siRNAs in MG132-treated HPF cells, SOD2, CAT or GPX siRNAs increased the number of Annexin V-stained cells. However, these siRNAs did not increase, but rather decreased ROS (DCF) levels in MG132-treated HPF cells. In addition, SOD1 and TXN siRNA, which did not enhance HPF cell death by MG132, strongly increased ROS (DCF) levels in these cells. CAT and GPX siRNAs attenuated O_2_^•−^ levels in MG132-treated HPF cells. Furthermore, SOD2 siRNA slightly increased O_2_^•−^ levels in MG132-treated HPF cells, whereas TXN siRNA decreased the level in these cells. Therefore, the alterations of MG132-induced HPF cell death by antioxidant-related siRNAs are not correlated with the ROS changes induced by these siRNAs. Moreover, administration with SOD1, GPX or TXN siRNA increased ROS (DCF) levels in HPF control cells, but SOD2 or CAT siRNA did not. None of these siRNAs increased O_2_^•−^ levels in HPF control cells. Because a change in the generation or metabolism of ROS in the cells is influenced by various pro-oxidant or antioxidant enzymes as well as activities in various cellular organelles, such as the mitochondria and the endoplasmic reticulum, our results suggest that the downregulation of each antioxidant protein by its corresponding siRNA does not simply increase ROS levels in HPF cells and can individually affect different ROS levels. Therefore, the effects of ROS level alterations induced by antioxidant-related siRNAs in MG132-treated HPF cells on cell death need to be further studied.

The redox status of cellular GSH is a crucial regulatory element in the protein ubiquitination system (34). GSH depletion due to proteasome inhibitors can lead to cell death (18,19). Likewise, MG132 dose-dependently increased the number of GSH-depleted cells in the HPF cells. BSO as a GSH synthesis inhibitor increased the numbers of GSH-depleted cells in MG132-treated HPF cells. However, 10 μM BSO showing a cell death effect in HPF control cells did not induce GSH depletion. Other reports definitely show that 100 μM or 1 mM BSO decreased GSH levels in MCF breast cancer cells (35) or U937 leukemia cells (36). These data imply that BSO differently influences GSH levels depending on the cell types or the incubation doses. In addition, although it is known that NAC containing a thiol group is a GSH precursor, NAC used in this study did not seem to be a GSH precursor since NAC did not affect GSH depletion in MG132-treated HPF cells. However, because we demonstrated that NAC significantly prevented GSH depletion in propyl gallate-treated HeLa cells (26), it is considered that NAC can be a GSH precursor or not depending on the co-incubated agents or cell lines. Moreover, all the siRNAs of antioxidant-related proteins did not influence GSH depletion in HPF control cells. These siRNAs except for TXN siRNA did not affect the number of GSH-depleted cells in MG132-treated HPF cells. Therefore, the downregulation of antioxidant proteins by their targeting siRNAs seems to not strongly alter GSH levels in HPF cells. Because TXN as a potent antioxidant can stimulate cell proliferation or may confer resistance to anticancer drugs (37,38), the downregulation of TXN may render cells sensitive to several cytotoxic drugs. However, our results showed that TXN siRNA did not enhance HPF cell death by MG132 but prevented GSH deletion in MG132-treated HPF cells. Therefore, the mechanism of the TRX siRNA-induced effects on the prevention of GSH depletion rather than on the enhancement of cell death in MG132-treated HPF cells needs to be further clarified. Taken together, our results suggest that the intracellular GSH levels seem to play a decisive role on MG132-induced HPF cell death, but changes of the content are not sufficient to predict cell death.

In conclusion, MG132 induced the growth inhibition and death of HPF cells, which were accompanied by increasing ROS levels and GSH depletion. The changes of ROS or GSH levels by NAC, vitamin C or BSO appeared to affect cell growth inhibition and death in MG132-treated HPF cells. In addition, administration of antioxidant-related siRNAs did not affect cell death, or ROS and GSH levels in MG132-treated or MG132-untreated HPF cells. Our present data provide useful information for understanding the cytotoxic or toxicological effects of MG132 in normal lung cells in relation to ROS and GSH levels.

## Abbreviations

HPF: human pulmonary fibroblast
MG132: carbobenzoxy-Leu-Leu-leucinal
ROS: reactive oxygen species
MMP (ΔΨ_m_): mitochondrial membrane potential
NADPH: nicotine adenine diphosphate
XO: xanthine oxidase
SOD: superoxide dismutase
CAT: catalase
GPX: GSH peroxidase
TXN: thioredoxin
FBS: fetal bovine serum
PI: propidium iodide
H_2_DCFDA: 2′,7′-dichlorodihydrofluorescein diacetate
DHE: dihydroethidium
GSH: glutathione
FITC: fluorescein isothiocyanate
CMFDA: 5-chloromethylfluorescein diacetate
MTT: 3-(4,5-dimethylthiazol-2-yl)-2,5-diphenyltetrazolium bromide
siRNA: small interfering RNA
NAC: N-acetyl cysteine
BSO: L-buthionine sulfoximine
